# Rapid Diagnosis of Acute Type A Aortic Dissection Through Multimodality Imaging

**DOI:** 10.1016/j.case.2024.02.007

**Published:** 2024-04-11

**Authors:** Chris Soudah, Abe DeAnda, Amer Abdulla

**Affiliations:** a John Sealy School of Medicine at University of Texas Medical Branch, Galveston, Texas; b Department of Thoracic Surgery, University of Texas Medical Branch, Galveston, Texas; c Department of Cardiology, University of Texas Medical Branch, Galveston, Texas

**Keywords:** Imaging, Acute coronary syndrome, Acute aortic syndrome, Dissection

## Abstract

• TAAD is a rare and potentially fatal disease.
• Aortic dissections have a wide range of common risk factors.
• This condition can mistakenly present similar to other acute coronary syndromes.
• Clinicians need clinical expertise and proper multimodality imaging for diagnosis.
• Misdiagnosis of this condition can be dangerous and fatal to patients.

## Graphical abstract

**Figure:**
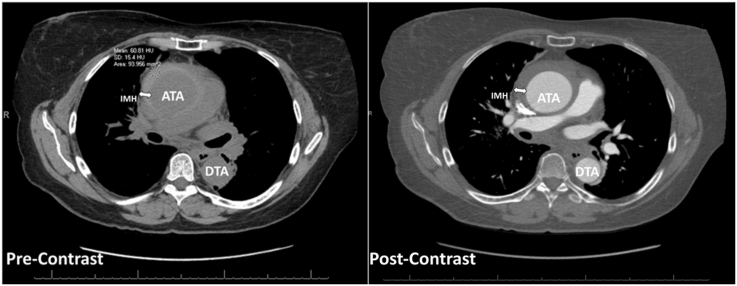

## Introduction

Aortic dissection is described as a tear in the inner smooth muscle layer of the aorta. It can be categorized into 2 types depending on which portion of the aorta is involved. Type A aortic dissections (TAADs) involve the ascending aorta and are the most common and dangerous type. These dissections typically present acutely in patients and involve more serious symptomology on presentation, such as dyspnea and sudden severe chest pain. This type is more often treated surgically. Complications include cardiac tamponade, acute severe aortic regurgitation (AR) leading to heart failure or shock, and acute coronary syndrome secondary to extension of the dissection into the coronary arteries, with all of these conditions being potentially fatal.^1^ We report a successful case that illustrates the diagnosis and management of TAAD with progression to intramural hematoma (IMH).

## Case Presentation

**Figure 1:**
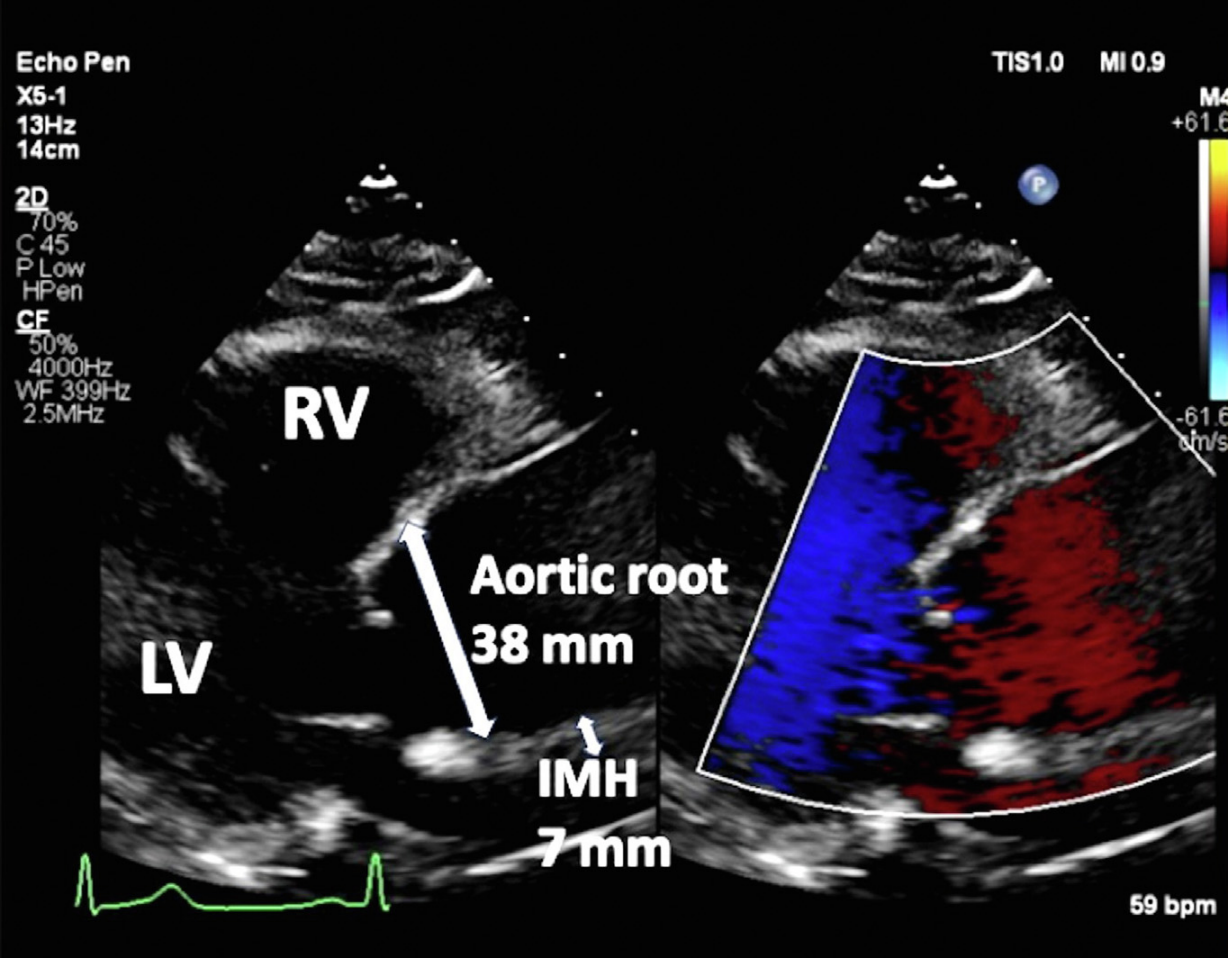
Two-dimensional TTE, parasternal long-axis view without (*left*) and with (*right*) color-flow Doppler in diastole, demonstrates a normal sized, 38 mm aortic root (*long arrow*) with abnormal aortic wall thickening consistent with the 7 mm IMH (*small arrow*; this was only noted on retrospective review of these images). There is no false lumen or aortic dissection flap seen. *RV*, Right ventricle.

**Figure 2:**
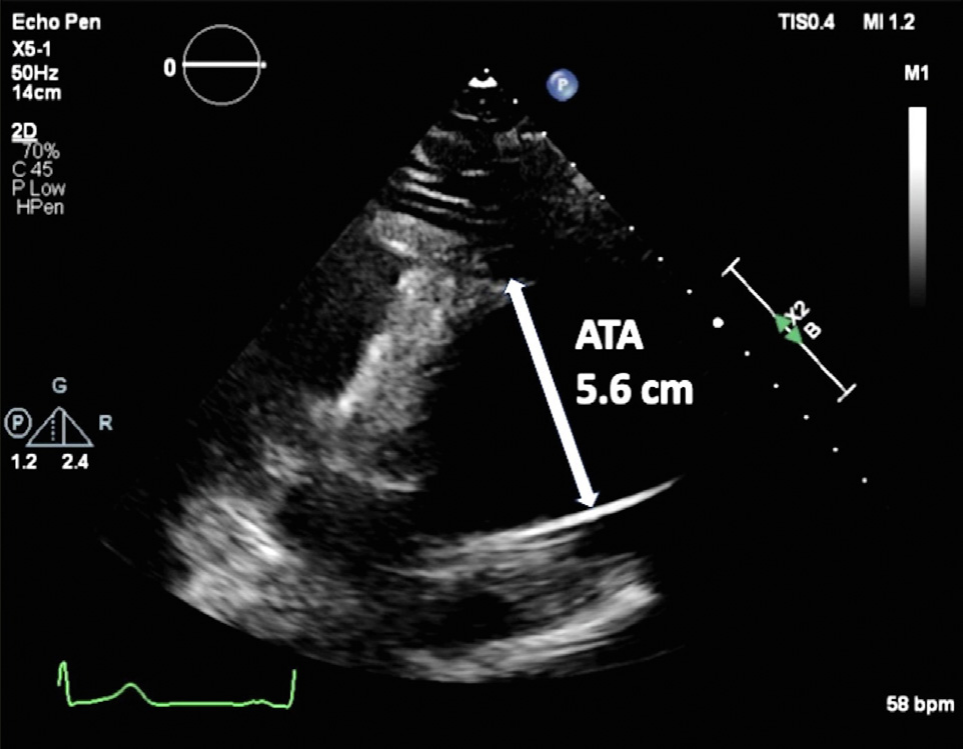
Two-dimensional TTE, modified parasternal long-axis view in diastole, demonstrates the dilated proximal ascending thoracic aorta (*arrow*) measuring 5.6 cm. *ATA*, Ascending thoracic aorta.

**Figure 3:**
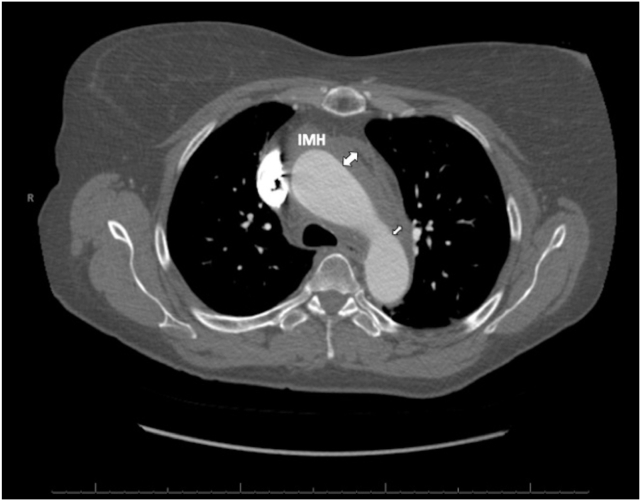
Contrast-enhanced CT scan, axial display at the aortic arch, demonstrates the superior extent of the IMH (*arrows*).

**Figure 4:**
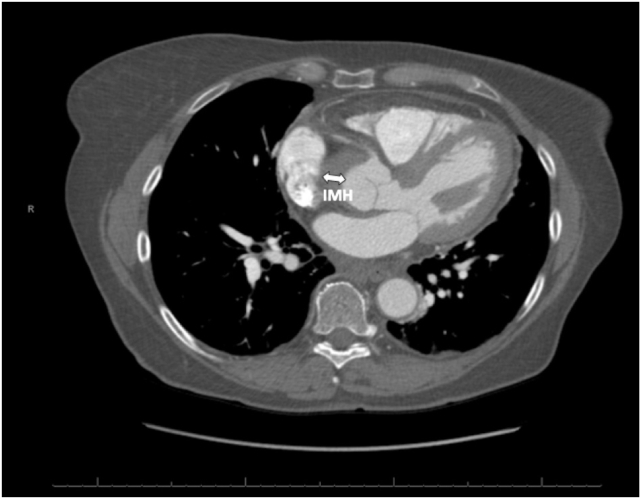
Contrast-enhanced CT scan, axial display at the sinus of Valsalva, demonstrates the proximal location of the IMH (*arrow*) adjacent to the origin of the right coronary artery.

**Figure 5:**
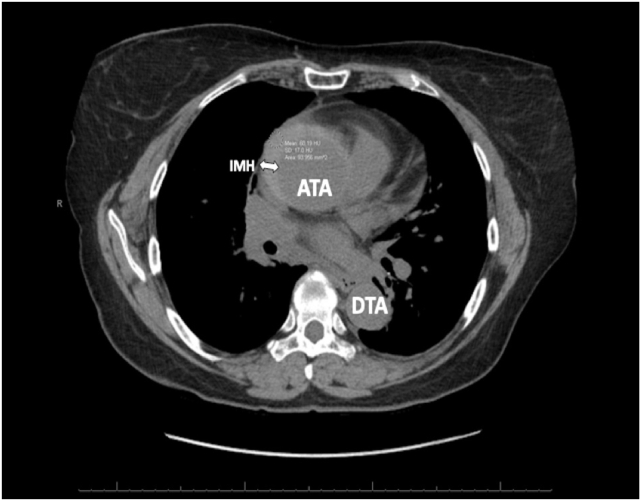
Nonenhanced CT scan, axial display at the level of the proximal ascending thoracic aorta, demonstrates a crescentic, high-attenuation (relative to the aortic lumen) region of aortic wall thickening that measures 60 Hounsfield units and strongly suggests an IMH (*arrow*). *ATA*, Ascending thoracic aorta; *DTA*, descending thoracic aorta.

**Figure 6:**
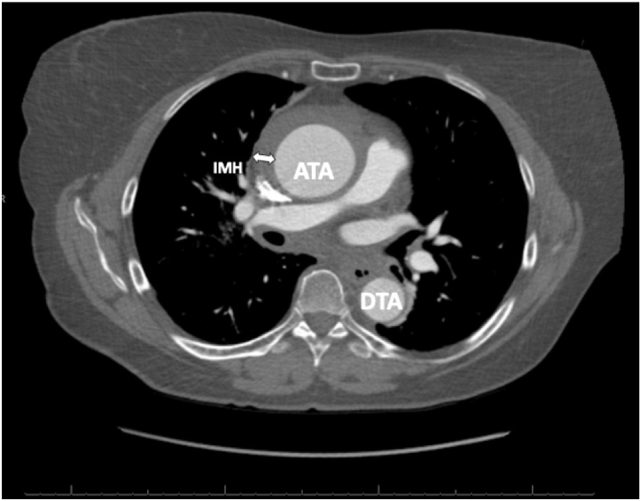
Contrast-enhanced CT scan, axial display at the proximal ascending thoracic aorta, demonstrates the IMH (*arrow*) as an unenhanced, crescentic, low-attenuation (relative to the aortic lumen) region of the aortic wall thickening with no evidence of an aortic dissection or intimal flap at this level. *ATA*, Ascending thoracic aorta; *DTA*, descending thoracic aorta.

A 67-year-old woman with a medical history of hypertension presented with severe substernal chest pain that began acutely the night before with an episode of emesis. The pain persisted throughout the night, prompting the patient to seek emergency care the following morning. Upon admission to the emergency department, the patient’s vital signs included a blood pressure of 143/83 mm Hg, pulse of 62 beats per minute, respiratory rate of 16 breaths per minute, and oxygen saturation of 100% on room air. Cardiovascular examination demonstrated a normal rate and regular rhythm, normal pulses, and normal heart sounds. Pulmonary examination findings showed normal pulmonary effort and normal breath sounds. An electrocardiogram revealed normal sinus rhythm without any acute ischemic changes. Troponin was not elevated at 0.011 ng/mL. Transthoracic echocardiogram (TTE) demonstrated normal biventricular size and function with no regional wall motion abnormalities and a severely enlarged proximal ascending aorta measuring 5.6 cm in diameter with normal diameter aortic root (Figures 1 and 2, Video 1), mild AR (Video 2), and no pericardial effusion. Computed tomography angiogram (CTA) of the chest revealed a type A IMH extending from the sinus of Valsalva to the aortic arch (Figure 3, Figure 4, Figure 5, Figure 6), just distal to the left subclavian artery origin, as well as an ascending aortic aneurysm measuring 6.5 by 6.1 cm including the IMH, with no obvious dissection flap. All coronary arteries appeared patent. The patient was taken urgently to the operating room. Reported intraoperative transesophageal echocardiogram (TEE) findings (images not available) revealed an ascending aortic aneurysm with a large IMH in the anterior wall. The aneurysm measured up to 6.3 cm including the hematoma. There was severe AR, with pressure half time (PHT) of 116 ms. The AR was secondary to the aortic dilation. There was no aortic stenosis. There was normal biventricular size and function. The patient underwent urgent ascending aorta repair with a hemiarch graft and suspension of the aortic valve (AV) with a 26 mm tube graft. Postoperative TEE imaging showed successful ascending aorta replacement with no change in left ventricular function and no wall motion abnormalities. The AR improved to mild in severity, and there was no pericardial effusion. Pathologic examination confirmed a large IMH in the setting of a thoracic aortic aneurysm with partial dissection and a small tear noted at the ST junction.

## Discussion

Type A aortic dissection is a rare and potentially fatal disease associated with aortic aneurysms that requires a high level of suspicion and prompt imaging to establish the diagnosis. A population-based epidemiologic study estimates the incidence of TAAD to be 2.2 in 100,000 compared to type B aortic dissections (TBADs), which occur in 1.5 of 100,000 individuals.^1^

In contrast to TAAD, TBADs involve the descending aorta and are most often seen chronically in patients, rarely causing life-threatening side effects. Given the chronic presentation of TBADs, this type is often treated with medical management and monitoring, utilizing medications that decrease blood pressure and heart rate. While this condition is usually not as life-threatening as TAADs, complications from TBADs can result in aortic rupture, branch artery occlusion with malperfusion, extension of the dissection, refractory pain, and aortic enlargement.^1^

Risk factors for aortic dissections are extensive and include older age, tobacco smoking, male sex, uncontrolled blood pressure, atherosclerosis, aortic aneurysms, and a number of genetic diseases that affect connective tissue.^2^ Given the fatal complications of this diagnosis, healthcare professionals must have clinical expertise and knowledge of which imaging modality to use in order to rapidly identify dissection and prevent delays in immediate treatment. The differential for aortic dissections includes other acute aortic syndromes (IMH, penetrating aortic ulcer, intimal tear without hematoma, and periaortic hematoma), acute coronary syndrome, pulmonary embolism, spontaneous pneumothorax, and esophageal rupture. The incidence of initial misdiagnosis of acute aortic syndromes is reported at 39%, which can result in delays in appropriate treatment.^3^ These patients are often assumed to be presenting with acute coronary syndrome and thus receive antiplatelet agents and anticoagulants, which may result in harm due to bleeding.^3^

One important factor to consider when reviewing cases of aortic aneurysms and dissections is the morphology of the AV. The AV is typically made up of 3 cusps: the left coronary cusp, the right coronary cusp, and the noncoronary cusp. The patient presented in this case had a tricuspid AV. Patients with bicuspid AVs are at an increased risk of aneurysm formation, with an incidence of 84.9 cases per 10,000 patient-years, and aortic dissection, with an incidence of 3.1 cases per 10,000 patient-years.^4^

Aortic aneurysms are also associated with several heritable thoracic aortic diseases (Marfan syndrome, Loeys-Dietz syndrome, vascular Ehler's-Danlos syndrome), other congenital diseases (Turner syndrome, coarctation of the aorta, tetralogy of Fallot), and pathogenic variants found in multiple genes (ACTA2, MYH11, PRKG1).^5^ It is estimated that approximately 20% of those with either a thoracic aortic aneurysm or aortic dissection have a family history of thoracic aortic dissection with at least 1 affected first-degree family member.^6^ This has been further supported with population studies illustrating a stronger link between familial cases in contrast to sporadic instances.^6^ Given this strong association, it is recommended that patients with either aortic aneurysms or dissections have first-degree family members screened with imaging modalities such as TTE given its affordability, availability, and convenience.^6^

Echocardiography plays a pivotal role in the diagnosis and management of aortic dissection. Transthoracic echocardiography can be performed rapidly and at bedside. Its sensitivity for detection of dissection is lower than other modalities, and it suffers from poor visualization of the distal ascending aorta, aortic arch, and descending aorta. However, it can be very helpful in detecting complications of dissection, such as tamponade, AR, and wall motion abnormalities associated with acute coronary syndrome (in conjunction with electrocardiograms and serial troponin measurements). For our patient in this report, TTE findings provided visualization of an enlarged proximal ascending aorta but lacked sensitivity to detect the IMH later seen on CTA (and in retrospect, noted on the TTE; Figure 1). Transesophageal echocardiography can also be performed relatively rapidly and at bedside in hemodynamically unstable patients and has a higher sensitivity and specificity for detecting dissection, due to its higher spatial resolution than that of TTE. It is also useful for assessing complications of dissection such as AR (with enhanced visualization of individual leaflets), tamponade, and wall motion abnormalities. Color-flow Doppler can be helpful in assessing for dissection flaps and identifying the false lumen. Although the AR was reported as severe due to the short PHT <200 ms seen on the intraoperative TEE, the use of this semiquantitative Doppler finding in acute AR should be cautioned. Since the velocity rate of the regurgitant jet is dependent on both the severity of the AR and, importantly, the compliance of the receiving chamber (the left ventricle [LV]), the correlation of the PHT only to the AR grade is often inaccurate in patients with normal or small LV chambers, history of hypertension, and LV hypertrophy. In the clinical setting of acute AR, these patients have impaired LV compliance and the LV pressure rises rapidly, causing the rate of pressure decline in the AR (e.g., the PHT) to decrease rapidly, resulting in a steep slope of the Doppler spectral tracing. Therefore, unlike severe chronic AR, where the dilated LV fills rapidly from the large regurgitant AR volume creating a short PHT, the short PHT in acute AR is more a result of the LV compliance than the AR volume. In our patient, this physiologic concept may have contributed to the discrepant findings of mild AR noted with color-flow Doppler compared with the very short PHT.

The use of ultrasound-enhancing agents has been shown to significantly increase the sensitivity and specificity of aortic dissection detection, particularly in TTE but also in TEE, by facilitating localization of the intimal tear and more easily identifying the true and false lumen.^7^ Three-dimensional TEE has been shown to enhance quantification of entry tear size, help define the morphology of a dissection, and better characterize the severity and mechanism of associated AR.^8^ Limitations to TEE include the necessity for sedation, a higher level of technical expertise required to attain adequate images, and limited visualization of the distal ascending aorta and proximal aortic arch.

In hemodynamically stable patients, computed tomography (CT) remains the imaging modality of choice due to its high sensitivity and specificity for acute aortic syndromes. Classically, dissection appears as a linear or curvilinear low-attenuation intraluminal mass, often associated with an aortic aneurysm. Electrocardiogram gating reduces the risk of motion artifact in the aortic root and ascending aorta, which are adjacent to the beating heart and may undergo significant translational motion.^9^ This artifact can result in the false appearance of a dissection flap.^9^ Electrocardiogram gating also allows for better definition of the AV morphology and function, more accurate measurements of aortic dimensions, assessment of cardiac function throughout the cardiac cycle, assessment of the patency of the coronary arteries, and a better appreciation of the dynamic motion of a dissection flap.^9^ Complete thrombosis of the false lumen can result in a crescentic or circular high-attenuation appearance along the aortic wall that does not enhance with contrast, as is characteristic of a true IMH.^9^ Comparison of CT noncontrast imaging with contrast imaging aids in the diagnosis of IMH. Noncontrast imaging demonstrates a crescentic, high-attenuation (relative to the aortic lumen) region of aortic wall thickening (Figure 5), typically around 50 to 70 Hounsfield units. On contrast imaging (Figure 6), IMH does not enhance, and appears lower in attenuation relative to the contrast-enhanced aortic lumen, with no dissection flap evident as seen with aortic dissection. If the IMH is small, it can be missed on contrast imaging, further emphasizing the importance of comparison with noncontrast imaging.^10^ A pitfall to be aware of with serial CT imaging is that of contrast third spacing into a nonbloody pericardial or pleural effusion.^11^ A follow-up scan may show hyperattenuation of these spaces, which may lead the provider to falsely conclude there is active bleeding into these spaces.^11^ This phenomenon can be recognized by the fact that third-spaced contrast tends to be more homogenously hyperattenuated than blood and tends to be in multiple compartments.^11^

Cardiovascular magnetic resonance imaging is also very sensitive and specific but rarely used as CT is usually more readily available. In addition, cardiovascular magnetic resonance imaging has no radiation exposure and is preferable in cases of serial imaging.^5^ Serial imaging is particularly helpful in evaluating the rate of growth of an aneurysm, with more rapidly growing aneurysms portending higher risk of rupture. Enlargement of the aortic root or ascending aorta by ≥0.5 cm in 1 year or ≥0.3 cm per year in 2 consecutive years in sporadic aneurysm cases or ≥0.3 cm in 1 year in heritable thoracic aortic disease or bicuspid AV syndromes is an indication for surgery.^5^ Cardiovascular magnetic resonance imaging is also exceptional in quantifying AR.^5^ Histopathologic examination of the resected segment of aorta is essential to confirm the diagnosis.

Regarding our patient, utilization of both TTE and CTA imaging modalities allowed for rapid identification of the dilated proximal ascending aorta and type A IMH. As the CTA showed IMH but no evidence of dissection, we believe the IMH had progressed from the time imaging was done until surgery, when an intimal dissection of the aorta was observed. Type A IMH cases are often managed as surgical emergencies due to the possibility of dissection adjacent to the critical structures of the coronary arteries and the AV. There are several high-risk features from imaging that favor emergent surgery, such as the extent of wall thickness, the presence of an ulcer, and significant AR. Our patient had both a significantly thickened wall and an ulcer. The TTE, however, revealed only mild AR.

## Conclusion

While TAADs are a rare and uncommon condition, they remain a dangerous and potentially fatal diagnosis, requiring immediate diagnosis with the use of multimodality imaging and expeditious surgical intervention. Given its similar presentation to other more common acute chest syndromes such as acute coronary syndrome, it is important to have a high index of suspicion for aortic dissection and utilize multimodality imaging to expeditiously establish the diagnosis and prevent delays in management.

## Ethics Statement

The authors declare that the work described has been carried out in accordance with The Code of Ethics of the World Medical Association (Declaration of Helsinki) for experiments involving humans.

## Consent Statement

The authors declare that since this was a non-interventional, retrospective, observational study utilizing deidentified data, informed consent was not required from the patient under an IRB exemption status.

## Funding Statement

This research did not receive any specific grant from funding agencies in the public, commercial, or not-for-profit sectors.

## Disclosure Statement

The authors do not have any relevant disclosures for this publication.

## Supplementay Data

Video 1 Two-dimensional TTE, parasternal long-axis view, demonstrates the severely dilated proximal ascending aortic aneurysm without pericardial effusion and with normal biventricular size and function. Note the proximal ascending aortic wall thickening proven on CT and surgery to represent a large IMH.

Video 2 Two-dimensional TTE, parasternal long-axis view without (*left*) and with (*right*) color-flow Doppler, demonstrates mild secondary AR with normal appearing AV.
